# Large language models in extracting key information from ICU patient text records from an Irish population: Comment

**DOI:** 10.1186/s40635-024-00678-9

**Published:** 2024-11-05

**Authors:** Hinpetch Daungsupawong, Viroj Wiwanitkit

**Affiliations:** 1 Private Academic Consultant, Phonhong, Lao People’s Democratic Republic; 2 Saveetha Medical College and Hospital, Saveetha institute of medical and Technical Sciences, Chennai, India

Dear Editor,

 “A pilot feasibility study comparing large language models in extracting key information from ICU patient text records from an Irish population” is an interesting article [1]. This study used anonymized health records to assess the performance of large language models (LLMs) in creating short ICU discharge summaries. Three LLMs were used: ChatGPT, GPT-4 API, and Llama 2. The study's goal was to identify the most effective stimulus structure for rapidly recording key clinical occurrences. Evaluation indicators included the model's capacity to detect significant events, as well as the readability and organization of the overall summary, which provided a full overview of the findings. The results showed that GPT-4 API outperformed ChatGPT and Llama 2, indicating that it has the potential for more accurate clinical synthesis.

Despite the promising outcomes, this technique has significant flaws. First, the training and validation used a relatively small dataset of only five ICU episodes for model construction, which may restrict the generalizability of the findings. Providing more information about the precise aspects of these symptoms may aid in determining whether they are typical or abnormal in the ICU. Furthermore, the evaluation stage entailed describing only six ICU symptoms, raising questions regarding the reliability of the performance metrics, particularly given the diversity in clinical context and presentation upon ICU admission. The small sample size may unintentionally bias the results, particularly if specific clinical occurrences are overrepresented. To further future research, this study could benefit from examining a larger and more diversified dataset that encompasses a wide range of situations and clinical circumstances. Expanding the range of warnings used in various groups and therapeutic settings could provide a more universal measure of effectiveness. Furthermore, using a more comprehensive assessment set that includes a broader panel of critical care experts could improve the strength of the recommendations in the conclusions and provide a more detailed understanding of the model's strengths and weaknesses in the clinical setting.

A longitudinal study design could assist evaluate progress over time and test the model's adaptation to changing clinical language and terminology. An innovative approach in this field could be the development of hybrid models that combine LLM skills with clinical decision support systems to ensure that results remain clinically relevant while limiting the possibility of hallucination. Incorporating real-time feedback mechanisms may enable these models to continuously learn from new forms of clinical data. Furthermore, investigating the relationships between LLM results and structured clinical data, such as electronic health records, may increase the quality of synthesis. Ethical impact assessments, formulating findings from a patient-centered perspective, and defining best practices for clinical application could all contribute to the advancement of this budding research field information from an ICU patient.

## Data Availability

There is no new data generated.
